# Incidence of anxiety and depression in a predominantly HIV-infected population with severe adverse drug reactions

**DOI:** 10.1186/2045-7022-4-S3-P95

**Published:** 2014-07-18

**Authors:** Eddy Zitha, Bonga Chiliza, Rudzani Muloiwa, Rannakoe Lehloenya

**Affiliations:** 1 Division of Dermatology Department of Medicine, University of Cape Town and Groote Schuur Hospital, South Africa; 2 Department of Psychiatry, University of Stellenbosch and Tygerberg Hospital, South Africa; 3 Department of Paediatrics and Child Health, University of Cape Town and Red Cross Children's Hospital, South Africa; 4 TBC

## Background

Little is known on the short-term or medium-term psychological and psychiatric sequelae following Stevens Johnson syndrome (SJS), toxic epidermal necrolysis (TEN) and drug reaction with eosinophilia and systemic symptoms (DRESS). Based on this we did a prospective study designed to assess anxiety and depression in patients with severe cutaneous adverse drug reactions by indicating higher Hospital anxiety and depression scale (HADS).

## Methods

We prospectively assessed 46 consecutive admissions with SJS, TEN and DRESS at a tertiary hospital in South Africa at 6 weeks and 6 months post discharge from hospital. We used a validated scoring system Hospital anxiety and depression scale (HADS) to assess anxiety and depressive in this cohort.

## Results

Forty-six patients were seen at six weeks and (n=38) 83% were reviewed at 6 months. Seventy-six percent of the participants were females and 82% were HIV-infected. Anxiety and depression were diagnosed in 37% and 30% respectively with six weeks with 8.6% exhibiting mixed anxiety and depressive symptoms. In comparison, at six months 13% had anxiety, 55% were depressed and 18.4% showed mixed anxiety and depressive symptoms. Nine and eight percent respectively at six weeks and six months warranted referral to a psychiatrist. Twenty-five percent of patients had anxiety and 40% had depression throughout the whole six months. The incidence of anxiety and depression was significantly associated with severity of the drug reaction.

## Interpretation

SJS/TEN and DRESS are associated with anxiety and depression for at least 6 months. SJS/TEN showed higher degree of anxiety and depression compares to DRESS. Our findings should help to improve awareness of psychological impact of severe adverse skin reactions as these may impact on treatment compliance

